# Delaminated Tears of the Rotator Cuff: MRI Interpretation with Clinical Correlation

**DOI:** 10.3390/diagnostics13061133

**Published:** 2023-03-16

**Authors:** Jun-Ho Kim, Seul Ki Lee

**Affiliations:** 1 Department of Orthopaedic Surgery, Center for Joint Diseases, Kyung Hee University Hospital at Gangdong, Seoul 05278, Republic of Korea; 2 Department of Radiology, St. Vincent’s Hospital, College of Medicine, The Catholic University of Korea, Seoul 06591, Republic of Korea

**Keywords:** shoulder, rotator cuff, magnetic resonance imaging, delaminated tear, classification

## Abstract

(1) Background: A delaminated tear is described as a horizontal split in the tendon substance. This review summarizes the clinical and radiologic characteristics of delaminated tears of the rotator cuff. (2) Methods: Initial radiological characteristics of a delaminated tear include the horizontal component of a partial-thickness tear determined using magnetic resonance (MR) arthrography. As demonstrated using indirect MR arthrography, the tear gradually progresses to be defined as either horizontal intrasubstantial splitting of the bursal and articular layers or differential retraction of the bursal and articular layers. (3) Results: The existence of delaminated tears is a poor prognostic factor in functional and morphologic outcomes after the repair of rotator cuff tendons and many surgical techniques have been introduced to solve this problem. Although the presence of a delaminated tear does not affect the arthroscopic repair outcome, the presence of medium-to-large, retracted delaminated tears may be an adverse negative prognostic factor after single-row repair. (4) Conclusion: Advances in imaging and surgical techniques have improved the detection of delaminated rotator cuff tears. Preoperative identification of delaminated tears on magnetic resonance imaging is clinically important because tailored surgical repair techniques must be chosen for successful outcomes.

## 1. Introduction

Magnetic resonance imaging (MRI) of the shoulder joint is the preferred diagnostic method for evaluating rotator cuff tears [1,2,3,4,5,6]. These tears can occur in the bursal surface, the articular surface, or the tendon intrasubstance [7,8]. The latter is called a “delaminated tear” and is described as a horizontal split of the tendon substance [9,10]. A delaminated tear is located between the articular and bursal layers of the tendon, with or without articular or bursal side tears [11]. In some delaminated tears, intratendinous fibers separated by horizontal splitting are retracted to varying degrees [7,12,13]. Identifying the delaminated tear in preoperative MRI is important because arthroscopic detection is difficult when there are no accompanying articular or bursal side tears [8,10]. Thus, it is imperative to determine the precise location and size of the delaminated tears of the rotator cuff preoperatively. This review summarizes the clinical and radiologic characteristics of delaminated tears of the rotator cuff and discusses their treatment and clinical outcomes.

## 2. Clinical Characteristics

Delaminated tears are common, although their prevalence and clinical impact have been underestimated. The orthopedic literature reports a prevalence of 38% to 92% for delaminated tears of the rotator cuff, depending on the surgical technique and the definition of delaminated tears [14,15,16,17,18,19]. Many surgeons diagnose delaminated tears by confining the arthroscope to the posterior portal when performing cuff repair, and only 11% of the delaminated tears can be detected using a standard posterior arthroscopic portal [17,20]. The lateral viewing portal makes it easy to visualize the entire delaminated layer, and systematic placement of the arthroscope in the lateral portal during repair is recommended to diagnose delaminated tears [17]. The definition of delaminated tears varies and has been somewhat vague in previous studies. Sonnabend and Watson [16] defined a delaminated tear as a horizontal partial-thickness split of the tendon substance. MacDougal and Todhunter [18] defined this type of tear as edge fraying or cleavage of 5 mm or greater and included high-grade partial-thickness tears. According to Han et al. [17], a delaminated tear was defined as a significant horizontal intratendinous tear requiring treatment. Kwon et al. [21] defined delamination in a full-thickness tear as an identifiable edge tear between the articular and bursal surfaces of the torn tendon and delamination in a partial-thickness tear as a retraction of the articular or bursal surface of the tendon with an identifiable horizontal gap between the two surfaces.

## 3. Pathogenesis

The histological and biomechanical reasons for delaminated tears may be multifactorial [7]. Both Ellman [8] and Sonnabend et al. [9] described the degree of retraction of the articular surface layer fibers as greater than that of bursal surface fibers due to the different histological structures of the two layers. In the supraspinatus tendon, the bursal surface layer mainly comprises tendons that elongate under tensile load and resist rupture. However, articular surface fibers are thinner and intertwined in both the lateral and longitudinal directions [22]. Therefore, because of the difference in stress between the two layers, they do not stretch as easily and are more vulnerable to tensile loading, leading to intratendinous lamination [23].

**Figure 1:**
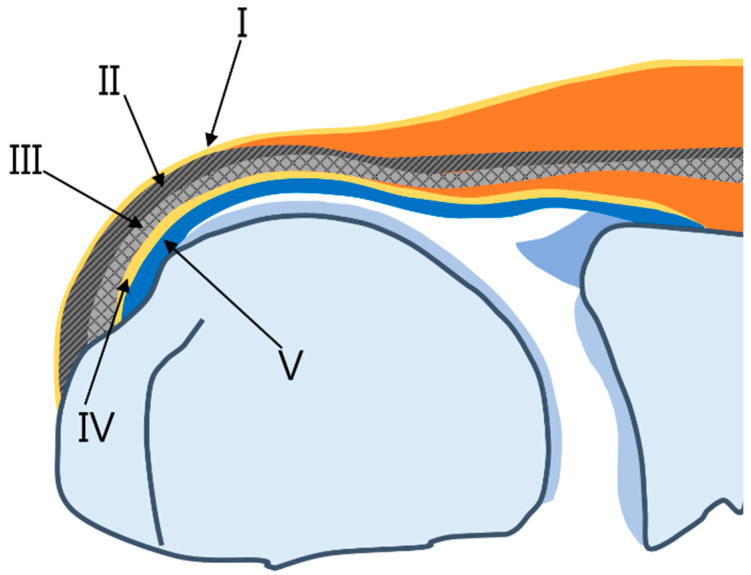
Schematic diagram of supraspinatus insertion with the histologic layers of the cuff in the coronal plane. Layer I is the most superficial layer, measuring 1 mm in thickness and composed of fibers from the coracohumeral ligament which overlay the cuff tendons. Layer II is composed of densely packed fibers that parallel the long axis of the tendon. Layer III is a deep tendon layer composed of smaller fascicles compared to layer II which are organized at an approximately 45 degree angle to the long axis of the tendon. Layer IV comprises loose connective tissue and thick collagen bands perpendicular to tendon fibers and merges with the coracohumeral ligament. Layer V represents the shoulder capsule.

**Figure 2:**
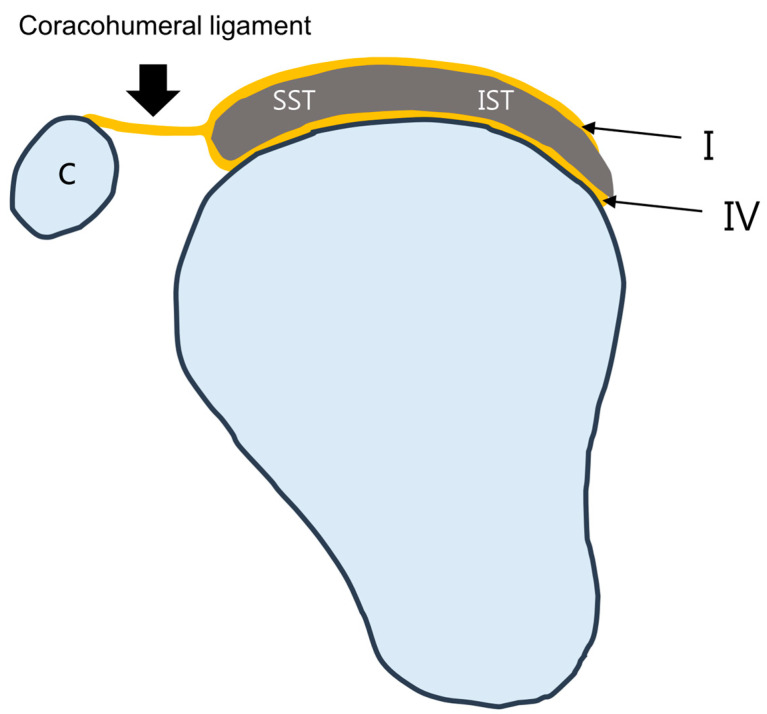
Schematic diagram of the coracohumeral ligament extension in the sagittal plane. The coracohumeral ligament has two extensions that course along the cuff tendons (layer I) and a deeper layer corresponding to the rotator cable (layer IV).

**Figure 3:**
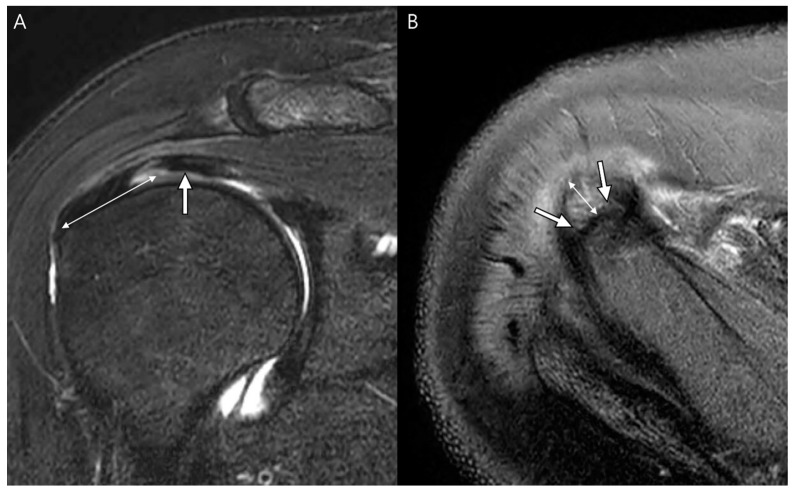
Rotator cable and crescent. (**A**) Oblique coronal fat-suppressed T2-weighted image and (**B**) axial fat-suppressed T2-weighted image demonstrate the low-signal thick bundle of rotator cable (thick arrow) perpendicular to the rotator cuffs and the distal fibers of rotator cuffs from the rotator cable to the greater tuberosity of the humerus called the rotator crescent (arrow bar).

The multilayered histological anatomy of the rotator cuff tendon may be associated with delaminated tears [10]. The microstructure of the rotator cuff tendons of the supraspinatus and infraspinatus is described as a five-layered structure (Figure 1) [24]. The first most superior layer is a thin (1 mm thick) superficial layer comprising the fibers of the coracohumeral ligament. The second is a thicker layer (3–5 mm) is parallel tendon fibers and the third layer is a deeper layer (3 mm) comprising tendon fibers without a uniform orientation that cross over one another at 45° angles. The fourth layer is a layer of loose connective tissue containing thick collagen bands, which fuse with the coracohumeral ligament along the anterior edge of the supraspinatus tendon; the fifth layer is thin (1.5–2 mm) and is formed by the capsule of the joint attached to the greater tuberosity by the Sharpey fibers. Sonnabend et al. [9] found that lamination splits often occur between layers of different collagen orientations (the second and third layers). The fourth layer runs perpendicular to the main fiber orientation of the cuff tendons. This layer contains a deep extension of the coracohumeral ligament (Figure 2) and has been described in many ways as a transverse band, pericapsular band, or rotator cable [24]. A thick bundle of fibers perpendicular to the supraspinatus tendon was called a rotator cable (Figure 3A), located about 1.4 cm from the footprint of greater tuberosity [25]. The distal fibers of the supraspinatus and infraspinatus extend laterally from the rotator cable and are inserted into the greater tuberosity of the humerus, and are called the rotator crescent (Figure 3B) [5].

Another gross anatomic and histologic analysis proposed an association between delamination and the infraspinatus tendon [19,26]. According to a previous study, the infraspinatus tendon could be divided into two components. First, the deep layer is thicker than the second superficial layer and corresponds to the articular capsule mixed with the infraspinatus muscle’s ventral oblique fibers which are more retracted and degenerated. The superficial layer corresponds to the dorsal transverse fibers of the infraspinatus mixed with the fibers of the supraspinatus tendon fibers. [19,26]. Nimura et al. [27] showed that the posterior upper capsule and its insertion were strongly associated with the deep layer of delaminated tears.

**Figure 4:**
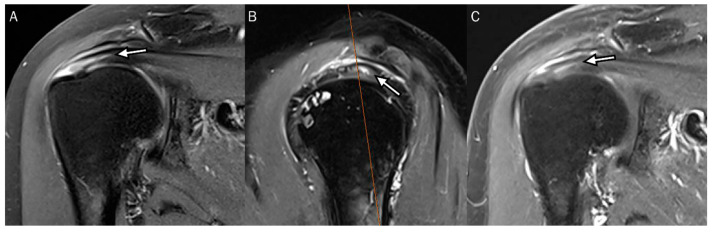
Enhancement of the synovial intraarticular tissue at the delaminated tear. (**A**) Oblique coronal fat-suppressed T2-weighted image and (**B**) coronal fat-suppressed T2-weighted image demonstrate the articular-delaminated full-thickness tear of the supraspinatus tendon with a visible cleavage (arrow). (**C**) The oblique coronal fat-suppressed T1-weighted enhanced image shows the synovial enhancement at the delamination (arrow).

Another study proposed that delamination reflects chronic degenerative changes in the tendon, which may be related to delamination [12]. FuKuda et al. [12] argued that intratendinous delaminated tears are caused by shear in the degenerated tendon. The regions of delamination contained hypovascularity related to hypertrophic changes in the small arteries of the tendon, and histological changes suggested that chronic degenerative changes play a role in the development of delaminated tears [9,22,23]. Furthermore, intratendinous delaminated tears may develop a synovial lining, propagate tears, and impede healing (Figure 4) [9]. Delamination can be a chronic condition resulting from repetitive and long-term strain mismatches between the two layers [9,21].

Although the delamination site remains unclear, Matsuki et al. reported that it frequently occurred at the posterior rather than at the anterior site of the torn site [28]. Regarding the normal layer structures of the rotator cuff tendons, Clark and Harryman reported that the rotator cuffs at the anterior part of the greater tuberosity comprise the musculotendinous portion of the rotator cuff, joint capsule, and the coracohumeral ligament. Note that they are closely intermingled [24]. Therefore, the connections between the layers appeared tighter in the front part of the tear than in the posterior part. This might be one reason the delamination was more in the posterior part than in the anterior part [29].

## 4. Radiologic Definition and Classification Using MRI

**Figure 5:**
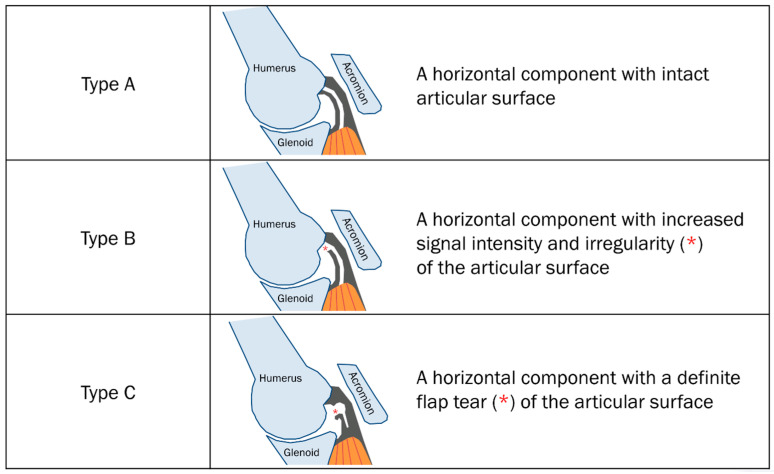
Schematic diagram of the types of delaminated tears of partial-thickness tears by Lee et al. [13].

The initial radiological characteristic determining the delaminated tear was the “horizontal component of partial-thickness tear” by Lee et al. [13]. This group used magnetic resonance (MR) arthrography in the abduction and external rotation (ABER) position [13]. The presence of a horizontal component in a partial-thickness tear was defined as the existence of linear intratendinous pooling of the contrast agent extended along the long axis of the rotator cuff [13]. The initial radiological classification using MR arthrography in the ABER position was limited to partial-thickness tears (Table 1) [13]. These tears were categorized by the articular surface shape and signal intensity of the rotator cuff on MR arthrography. Type A tears have a horizontal component with an intact articular surface. Type B tears have a horizontal component with increased signal intensity and irregularity of the articular surface. Type C tears have a horizontal component with an actual flap tear (torn edge) of the articular surface (Figure 5) [13].

Walz et al. [7] defined a delaminated tear as a horizontal retraction of either the bursal or articular surface, manifested as thickening of the torn retracted edge and/or interstitial horizontal splitting of the tendon. This tendon split was viewed as a fluid-like high signal intensity on fat-suppressed T2-weighted oblique coronal images and included both partial- and full-thickness tears.

**Figure 6:**
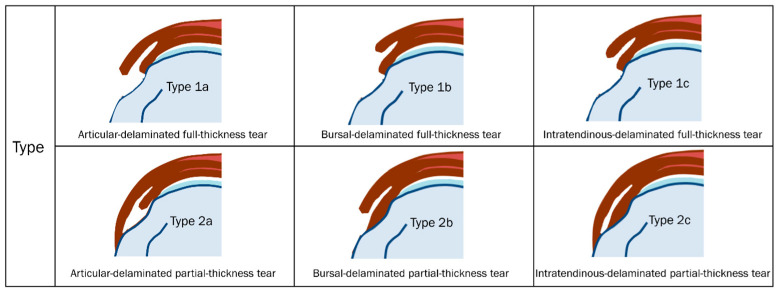
Schematic diagram of the types of delaminated tears of full- and partial-thickness tears by Choo et al. [11].

According to Choo et al. [11], a delaminated tear can be evaluated using indirect MR arthrography to determine an intratendinous horizontal split between the articular and bursal layers of the tendon regardless of whether there is a different degree of retraction between the two layers. The intratendinous horizontal splitting tear was displayed as a high signal intensity on fat-suppressed T1-weighted or T2-weighted imaging [11]. This group classified delaminated tears into six types according to the degree of tear (full-thickness or partial-thickness) and retraction degree of each layer (Table 2, Figure 6) [11]. Type 1a tears were articular-delaminated full-thickness tears; full-thickness tears in which the articular layers were more medially retracted than the bursal layers, regardless of intratendinous horizontal splitting tears. Type 1b tears were bursal-delaminated full-thickness tears; full-thickness tears in which the bursal layers were more medially retracted than the articular layers, regardless of intratendinous horizontal splitting tears. Type 1c tears were intratendinous-delaminated full-thickness tears; full-thickness tears in which the articular layers were equally retracted to the bursal layers and combined with intratendinous horizontal splitting tears. Type 2a tears were articular-delaminated partial-thickness tears; articular-surface partial-thickness tears with intratendinous horizontal splitting tears. Type 2b tears were bursal-delaminated partial-thickness tears; bursal-surface partial-thickness tears with intratendinous horizontal splitting tears. Type 2c tears were intratendinous-delaminated partial-thickness tears; isolated intratendinous horizontal splitting tears [11].

**Figure 7:**
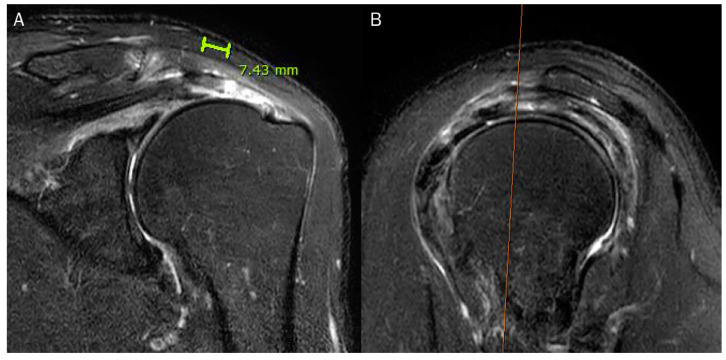
Arthroscopically proven full-thickness tear of the supraspinatus tendon without delamination. (**A**) Oblique coronal fat-suppressed T2-weighted image and (**B**) coronal fat-suppressed T2-weighted image demonstrate the full-thickness tear of the supraspinatus tendon with a visible cleavage measured at least 1 cm in length. The tendon was not classified as a delaminated tear.

According to Bierry et al. [30], a delaminated tear is defined as either horizontal intrasubstance splitting of the bursal and articular layers by a plane of fluid, or differential retraction of the bursal and articular layers regardless of an intervening plane of fluid between them. A tendon was not classified as having a delaminated tear if it exhibited a minimal differential retraction of 1 cm between bursal and articular layers or an intervening plane of fluid measured at least 1 cm in length (Figure 7) [30].

Collectively, the following preoperative morphological MRI findings have been reported for delaminated tears of the rotator cuff (Table 3): type of delaminated tear, retraction length of the articular and bursal layers, the length of the intrasubstance cleavage, the length of the anteroposterior tear, and fatty muscle infiltration of rotator cuffs [10,30,31,32].

## 5. Radiologic Diagnosis of Delaminated Tears Using MRI

Using MR arthrography in the ABER position, Lee et al. [13] reported that the prevalence of delaminated tears in the supraspinatus-infraspinatus tendons of the enrolled shoulders was only 3.9%. Flap tears (type C) (62%) were the most common horizontal component in partial-thickness rotator cuff tears, followed by articular surface irregularities (type B) (21%) and intact articular surfaces (type A) (17%) [13]. A possible mechanism that may lead to intratendinous pooling of a contrast agent in the horizontal component with an intact articular surface (type A) is the transport of contrast agent via a connection between the damaged rotator cuff tendons or absorption of the contrast agent by the rotator cuff, which has fraying and the friability of the tendon was not detected on MRI [13].

**Figure 8:**
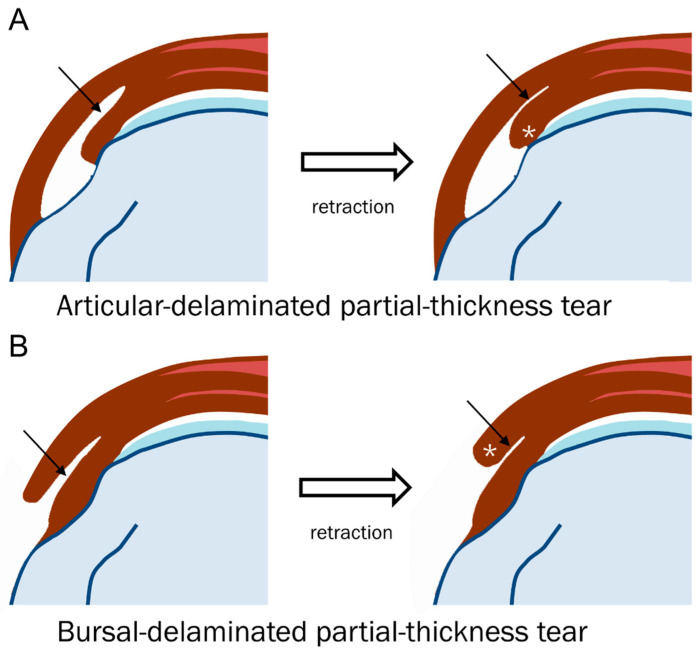
Schematic diagram of a delaminated tear with or without a visible cleavage. (**A**) At the initial, the articular-delaminated partial-thickness tear shows visible cleavage (arrow), and at retraction, it shows a retracted torn tendon (*) with uncovering cleavage (arrow). (**B**) At the initial, the bursal-delaminated partial-thickness tear shows visible cleavage, and at retraction, it shows a retracted torn tendon (*) with uncovering cleavage (arrow).

**Figure 9:**
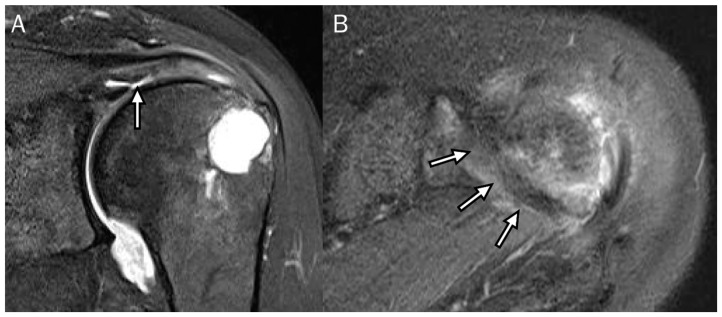
Articular-delaminated partial-thickness tear of the supraspinatus tendon with retraction (no visible cleavage). (**A**) Oblique coronal fat-suppressed T2-weighted image and (**B**) axial fat-suppressed T2-weighted image demonstrate an undersurface delaminated tear with subsequent retraction presenting as a retracted rotator cable (arrows).

Walz et al. [7] analyzed delaminated tears accompanying partial- and full-thickness tears using MRI without arthrography. They reported that delaminated tears occurred at the supraspinatus-infraspinatus tendon in only 1.8% of the enrolled shoulders [7]. Tears occur most often in partial-thickness tears of the supraspinatus tendon and always involve the articular surface of the tendon [7]. Only one case was accompanied by a full-thickness tear of the supraspinatus tendon, with retraction of the articular surface more medially than the bursal surface [7]. The group also reported that only half of the delaminated tears had a visible cleavage plane in the interstitium, indicating that if the torn edge was retracted and subsequently thickened, the cleavage plane was unnecessary for diagnosis (Table 4, Figure 8) [7]. When delaminated tears peel back along the plane of intrasubstance cleavage, the interstitial component is exposed and invisible [7]. The element of retraction manifested as thickening of the torn edge defines tears as a delaminated type (Figure 9) [7].

**Figure 10:**
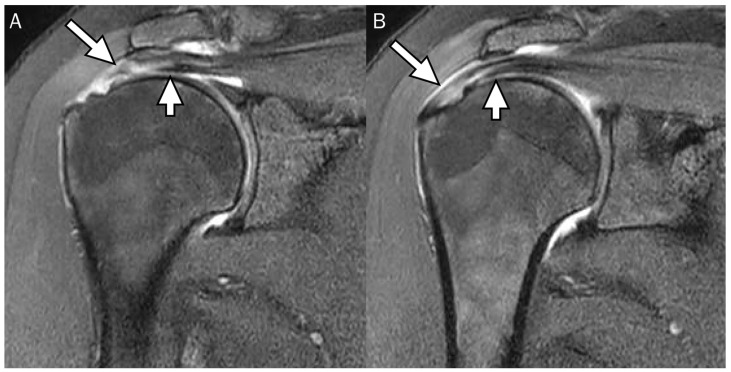
Articular-delaminated full-thickness and partial-thickness tear of the supraspinatus tendon. (**A**) Oblique coronal fat-suppressed T2-weighted image at the mid-portion of the supraspinatus tendon shows an articular-delaminated full-thickness tear with a visible cleavage and (**B**) subsequent posterior image of coronal fat-suppressed T2-weighted image demonstrates an articular-delaminated partial-thickness tear with a visible cleavage. Note that the articular surface (short arrows) is retracted more medially than the bursal surface (long arrows).

Using indirect MR arthrography, Choo et al. [11] found that the prevalence of delaminated tears in the supraspinatus-infraspinatus tendon was 23%. The most common types (71%) are articular-delaminated full-thickness tears (type 1a) and articular-delaminated partial-thickness tears (type 2a), which means that articular layers were constantly retracted more than the bursal layers (Figure 10). All types of delaminated tears usually occurred in the supraspinatus tendon [11]. If the delaminated tear length was defined as the sum of the medial-to-lateral extent and the medial-to-lateral degree of the retraction difference between the articular and bursal layers of the tendon, the bursal-delaminated full-thickness tear (type 1b) had the greatest length and the articular-delaminated partial-thickness tear (type 2a) had the smallest length [11].

**Figure 11:**
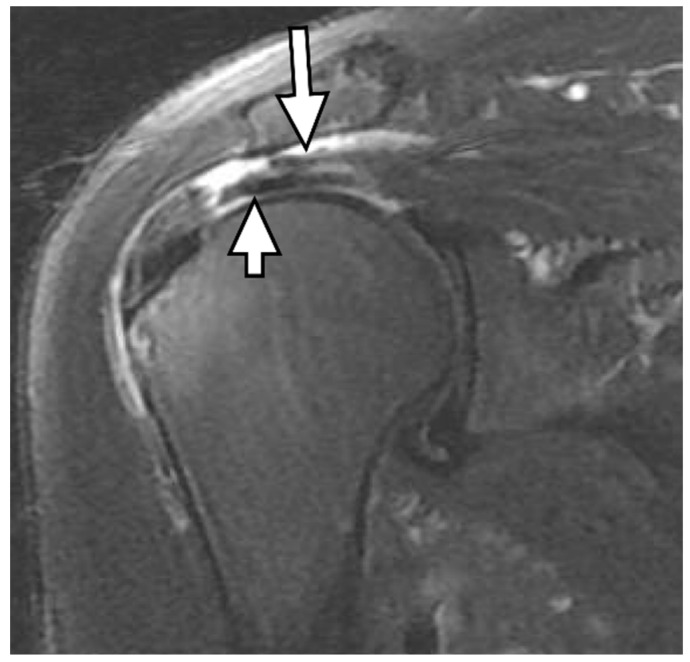
Bursal-delaminated full-thickness tear of the supraspinatus tendon. An oblique coronal fat-suppressed T2-weighted image of the supraspinatus tendon shows a bursal-delaminated full-thickness tear with a visible cleavage. The bursal surface (long arrow) is retracted more medially than the articular surface (short arrow).

Bierry et al. [30] reported that delamination was present in 13% of full-thickness rotator cuff tears. They studied the pattern of tendon retraction in patients with delaminated tears. The articular layer was always retracted beyond the bursal layers, except in one case of bursal-delaminated tears (Figure 11); these data correspond to orthopedic reports [16,18,19,30].

The gold standard for diagnosing tears of rotator cuff tendons is considered to be MRI [33]. MRI showed a low sensitivity of 35.5% and excellent specificity of 100%, with only 57.3% diagnostic accuracy for diagnosing delaminated tears in 349 shoulders [34]. A positive predictive value of 100% and a negative predictive value of 44.2% were reported [34]. Even if there is no delamination on MRI, retraction of the tendon with a higher amount of fatty muscle infiltration might also indicate the presence of tendon delamination [34]. Surgeons can benefit from the knowledge of tendon delamination by performing surgery more quickly for larger delaminated tears and shorter conservative treatment followed by surgery for smaller delaminated tears [30].

## 6. Treatment and Clinical Outcome

Several reports since 2005 agreed that the presence of delaminated tears is a negative prognostic factor in functional and morphologic results after the repair of rotator cuff tendons [14,15,16,29,32,35,36]. An improvement in biomechanical properties, improved clinical outcomes, and decreased retear rates have been reported for the repair of delaminated tears [31,37,38,39,40,41,42,43]. The intratendinous laminated component of rotator cuff tears should be repaired using a number of surgical techniques to restore normal biomechanics of the cuff and reduce the risk of repair failures [10]. These include two popular surgical techniques. The first is to use double-row repair techniques to suture each laminated layer separately by attaching the inner layer to the inner footprint and the outer layer to the outer footprint [10,44]. Another technique, “En bloc.”, is to suture both delaminated cuffs with a single stitch to join both layers and reduce shear force [10,45]. Curettage of the synovial lining of the opposing layers of the torn tissue is recommended before attempting repair [9]. A meta-analysis was conducted to compare the clinical outcomes of arthroscopic double-row repair and En bloc techniques. The results showed no statistical difference in outcomes between the two interventions for the University of California at Los Angeles (UCLA) score, constant score, visual analog scale (VAS) score, range of motion, and retear rate [46]. In Kim’s randomized controlled trial study, pain relief with a layered repair was more evident than that with a full-thickness repair in terms of VAS scores [40]. Ren et al. reported that layered suturing was considered to have a longer operation time and no significant difference in clinical efficacy than full-thickness suturing [47]. Cha et al. reported that optimal anatomical balance can be achieved based on the direction of contraction of the structure of each layer by layered repair [41].

Although the clinical significance of delamination has a negative prognostic effect on clinical and radiologic outcomes, several recent studies have reported that the presence of a delaminated tear does not affect the outcome when properly repaired [16,18,35,45,48]. MacDougal and Todhunter [18] compared clinical outcomes with delaminated and non-delaminated tears after the mini-open technique and reported significant improvement in postoperative scores in both groups; however, the differences were not statistically significant. Kim et al. [49] and Kwon et al. [21] also suggested that delaminated tears may not be independent prognostic factors when comparing delaminated and non-delaminated tears. However, Boileau et al. [20] reported that patients with chronic delaminated and retracted tears were at risk of failure after single-row repair, and patients with small delaminated tears involving only the supraspinatus could be treated with a single-row repair with a high chance of tendon healing. Collectively, the presence of medium to large retracted delaminated tears is a negative prognostic factor in anatomic outcomes after single-row repair and affects the rate of tendon healing [20,50,51,52,53]. Similarly, tendon delamination is increased in larger rotator cuff tears, and tear size is closely associated with fatty muscle infiltration [34,54]. Therefore, the repair of the double-layered rotator cuff is important to ensure optimal healing of the tendons and reduce the risk of retears [34,35]. Thus, arthroscopic double-layered repair is increasingly used in clinical practice to restore the original anatomy of the rotator cuff in the footprint area [46].

In arthroscopic-layered repair, the bursal and articular side inserts of the torn rotator cuff are separately fixed. This technique fits the original anatomy of the rotator cuff and facilitates the healing of the torn rotator cuff compared to any other repair [10]. In theory, Sugaya et al. found that the articular-side delamination tear of a rotator cuff originated from the oblique fiber behind the infraspinatus muscle, thicker than the transverse fiber derived from the combination of the supraspinatus and infraspinatus muscles. In addition, the articular side had greater contraction tension than the bursal side; therefore, double-layered repair provided better structural stability and functional restoration than conventional full-thickness repair [55]. Mochizuki et al. suggested that in the two-layer structure of the rotator cuff delamination tear, the articular side consists mainly of the articular capsule, stops inside the greater tuberosity, and the repair direction should be from the inner to the outer layer. The bursal side mainly consists of the infraspinatus muscle and stops in the front of the greater tuberosity and is repaired from the posterior to the front layer [51].

## 7. Conclusions

The different histological structures between the articular and the bursal layers of the tendon can explain delaminated rotator cuff tears. Improved imaging and surgical techniques have increased the detection of delaminated rotator cuff tears. Preoperative identification of delaminated tears on MRI is clinically important since the delaminated tears are regarded as a negative prognostic factor for clinical and radiological outcomes after arthroscopic rotator cuff repair, particularly when the tear is neglected. Thus, appropriately tailored surgical repair techniques must be selected for successful outcomes in patients with delaminated rotator cuff tears. An important feature of a delaminated tear is the increased retraction range of the articular layer relative to the bursal layer. Both arthroscopic full-thickness repair and double-layered repair involving the retracted articular layer have advantages for treating delaminated rotator cuff tears. Recent literature has demonstrated satisfactory clinical outcomes after repairing delaminated rotator cuff tears; therefore, surgeons should carefully identify these tears and consider tear characteristics on preoperative MRI when repairing delaminated rotator cuff tears.

## Figures and Tables

**Table 1:** Delaminated tear of partial-thickness tear by Lee et al. [13].

Type A	a horizontal component of a tear with an intact articular surface
Type B	a horizontal component with increased signal intensity and irregularity of the articular surface
Type C	a horizontal component with a definite flap tear (torn edge) of the articular surface

**Table 2:** Delaminated tears of full- and partial-thickness tears by Choo et al. [11].

Type 1	
Type 1a	articular-delaminated full-thickness tears
Type 1b	bursal-delaminated full-thickness tears
Type 1c	intratendinous-delaminated full-thickness tears
Type 2	
Type 2a	articular-delaminated partial-thickness tears
Type 2b	bursal-delaminated partial-thickness tears
Type 2c	intratendinous-delaminated partial-thickness tears

**Table 3:** Preoperative morphological MRI findings for delaminated tears of the rotator cuff.

1	type of delaminated tear
2	retraction length of the articular and bursal layers
	length of the intrasubstances cleavage
	length of the anteroposterior tear
3	fatty muscle infiltration of rotator cuffs

**Table 4:** Three components of delaminated tears on preoperative MRI.

Component 1	Component 2	Component 3
articular-delaminated	partial-thickness	visible cleavage
bursal-delaminated	full-thickness	no visible cleavage and thickened torn edge

## Data Availability

Not applicable.
